# Evaluation of Toll-Like Receptor 2 and 4 RNA Expression and the Cytokine Profile in Postmenopausal Women with Metabolic Syndrome

**DOI:** 10.1371/journal.pone.0109259

**Published:** 2014-10-17

**Authors:** Claudio Lera Orsatti, Eliana Aguiar Petri Nahas, Jorge Nahas-Neto, Fabio Lera Orsatti, Vanessa Innocenti Giorgi, Steven S. Witkin

**Affiliations:** 1 Department of Gynecology and Obstetrics, Botucatu Medical School, Sao Paulo State University-UNESP, Botucatu, São Paulo, Brazil; 2 Department of Sports Science Institute of Health Sciences-UFTM, Uberaba, Minas Gerais, Brazil; 3 Department of Microbiology and Immunology, Institute of Biosciences, Sao Paulo State University- UNESP, Botucatu, Sao Paulo, Brazil; 4 Department of Obstetrics and Gynecology, Division of Immunology and Infectious Diseases, Weill Cornell Medical College, New York, New York, United States of America; University of Padova, Italy

## Abstract

**Objective:**

To evaluate the gene expression of Toll-Like (TLR-2 and TLR-4) receptors and cytokine profile in postmenopausal women with or without metabolic syndrome (MetS).

**Methods:**

In this cross-sectional study, 311 Brazilian women (age≥45 years and amenorrhea≥12 months) were included. Women showing three or more of the following diagnostic criteria were diagnosed as positive for MetS: waist circumference>88 cm, triglycerides≥150 mg/dL, HDL cholesterol<50 mg/dL, blood pressure≥130/85 mmHg, and fasting glucose≥100 mg/dL. The expression of TLR-2 and TLR-4 in peripheral blood was evaluated by RNA extraction and subsequent real time PCR analysis. The cytokine profile, tumor necrosis factor alpha (TNF-α) and interleukins 1β, 6, and 10, were measured by ELISA.

**Results:**

The expression of TLR-2 RNA was demonstrated in 32.5% and TLR-4 in 20.6% of the subjects. There was no association between the expression of TLR-2 and TLR-4 and the presence or absence of MetS (*P*>0.05). A greater production of IL-6 was associated with TLR-2 and TLR-4 expressions and greater production of TNF-α was associated only with TLR-2 expression (*P*>0.05). Only the lower quartile of IL-10 was associated with the presence of the MetS (*P*>0.05).

**Conclusions:**

TLR-2 and TLR-4 expressions were associated with increased pro-inflammatory cytokines, IL-6 and TNF-α, with no association with biomarkers of MetS. The low concentrations of IL-10 may suggest an anti-inflammatory modulation in postmenopausal women with MetS.

## Introduction

Metabolic syndrome (MetS) is defined by a set of metabolic risk factors that include abdominal obesity, dyslipidemia, hypertension and dysglycemia [1]. It affects approximately 30% of the female population over 50 years with a threefold increase in the risk of mortality from cardiovascular disease (CVD) [2]–[4]. The syndrome is associated with a metabolic disorder dominated by insulin resistance (IR), in which the normal action of insulin is impaired. Abdominal obesity is a pro-inflammatory state that contributes to IR, a condition suggested as the cause of dyslipidemia, glucose intolerance and increased blood pressure [5], [6]. The MetS is associated with increased risk of developing atherosclerosis and coronary heart disease (CHD) in postmenopausal women [7].

In CHD, conventional metabolic risk factors present in the MetS, such as dyslipidemia, hypertension, diabetes, obesity, are common [8], but individual differences in the inflammatory profile may modulate the severity of the atherosclerotic process [9]. Atherosclerosis is defined as a chronic, progressive and systemic process, consequent to the inflammatory and fibroproliferative response caused by aggression to the endothelial surface of arteries [10]. It can be triggered by injury to the arterial endothelium attributed to factors such as hypertension, hyperinsulinemia, dyslipidemia, and mainly by the deposition of oxidized LDL molecules [11]. After the initial aggression, monocytes and T cells are recruited from peripheral blood to the arterial wall, and penetrate the intima of the vessels and phagocyte oxidized LDL, thus promoting the release of inflammatory cytokines [10]. Among these, interleukins 1 and 6 (IL-1 and IL-6) and tumor necrosis factor alpha (TNF-α) have a role in perpetuating the inflammatory response and modifying the surface to an anticoagulant endothelial prothrombotic state. IL-6 stimulates liver production of C-reactive protein (CRP), the latter being recognized as an acute phase protein in inflammatory processes [12], [13]. The elevation of serum CRP is considered an independent risk factor for atherosclerosis, particularly in women [12], besides predisposing to acute myocardial infarction (AMI), cerebrovascular accident (stroke), peripheral artery disease and sudden death [14].

The expression of pro-inflammatory cytokines occurs by activation of Toll-like receptors (TLRs), particularly TLR-2 and TLR-4 [15]–[17]. TLRs are transmembrane proteins characterized by a leucine-rich repeat (LRR) extracellular domain, and an intracellular Toll/IL-1R [18], [19]. They are divided according to their location in the cell. TLR-1/2/4/5/6/10 are expressed on the cell surface while TLRs-3/7/8/9 are in intracellular endosomes compartments [19], [20]. TLRs are widely expressed in immune, epithelial and endothelial cells [21]–[25]. TLR activation results in the induction of pro-inflammatory cytokines, phagocytosis, and an oxidative burst [26], [27]. TLRs can be activated by cells (leukocytes) on the cardiovascular system, showing a link between the development of CHD and the immune system, although the role of individual members of the TLR family in the pathophysiology of this disease still requires further investigation [2].

The cellular expression of TLRs in atherosclerotic lesions has been previously observed [16], [26], [28], [29]. The activation of TLR-2 plays a central role in the regulation of vascular inflammation in mice, suggesting that TLR-2 induces increased production of proinflammatory cytokines (TNF-α, IL-1β, and IL-6) and decreased formation of vascular tissue. These results were contrary to what was observed in mice deficient in TLR-4 [30]. Considerable evidence associates atherosclerosis with TLR signaling. Studies controversial showed that alterations polymorphic in TLR-2/4 receptor may or may not associate itself with in atherosclerosis and in CHD [2], [31], [32]. However, other researchers reported that TLR-4 polymorphism is not significant in relation to atherosclerosis and other coronary diseases [33], [34]. In another study, it was observed that a TLR-2 polymorphism was associated with CVD and not a polymorphism in TLR-4 [35].

There is little data on the expression of TLRs in postmenopausal women. The evaluation of TLRs can provide information about the regulation of inflammation in the endometrium [25] and in some types of cancer [36], besides identifying women with atherosclerosis, among those with low cardiovascular risk [37]. A recent study demonstrated that the expression and activity of TLR-2 and TLR-4 are increased in monocytes from patients with MetS compared to patients without MetS, in turn, these receptors are activated and produce pro-inflammatory cytokines contribute to the increased risk of CHD [38]. However, the literature is scarce regarding the pathway of inflammation in patients with MetS.

There is growing evidence that TLR expression and circulating inflammatory molecules influence the risk of CHD and that the detection and monitoring of inflammation play important roles in early diagnosis, particularly in postmenopausal women with MetS. The aim of this study was to evaluate and compare TLR-2 and TLR-4 gene expression and the cytokine profile in postmenopausal women with or without MetS.

## Methods

### Study Design and Sample Selection

This is a clinical, analytical, and cross-sectional study. The study population was postmenopausal women, aged 45–70 years, attending a public outpatient center in a University Hospital in Southeastern Brazil from February 2011 to June 2012. Sample size estimation was based on the study by Yasui et al. [39] that showed mean values of IL-6 in premenopausal women was 2.7 pg/ml vs. 1.6 pg/ml in postmenopausal women. Considering this difference, with a 5% level of significance and a 10% type-II error (90% test power), the need to evaluate at least 85 participants was estimated. Women whose last menstruation was at least 12 months prior to study initiation and age≥45 years old were included (n = 311). The exclusion criteria were: (1) known high cardiovascular risk due to existing or preexisting CHD, cerebrovascular arterial disease, abdominal aortic stenosis or aneurysm, peripheral artery disease, chronic kidney disease; (2) history of: hepatitis B and C, acute infection, lower genital tract infection, chronic inflammatory or autoimmune diseases (ulcerative colitis, Crohn's disease, rheumatoid arthritis, lupus, etc), cancer, and addiction to either alcohol or illicit drugs. Written informed consent was obtained from all participants and the study was approved by the Research Ethics Committee of Botucatu Medical School, Sao Paulo State University/UNESP.

### Methodology

During the consultation, all subjects underwent individual interviews in which the following data were collected: age, time since menopause, current smoking, use of hormone therapy (HT), personal history of hypertension, diabetes and physical activity, as well as family history of CHD (acute myocardial infarction in 1st degree relative male aged<55 years and females aged<65 years). Blood pressure was measured using a standard aneroid sphygmomanometer on the right arm with patients in the sitting position, forearm resting at the level of the precordium and the palm of the hand facing upwards, after a five-minute rest. Smokers were defined as persons who reported smoking regardless of the number of cigarettes smoked. Women who practiced aerobic physical exercise of moderate intensity for at least 30 minutes, five times a week (150/min/week) or resistance exercise three times a week were considered to be active [40]. Women showing three or more of the following diagnostic criteria proposed by the US National Cholesterol Education Program/Adult Treatment Panel III (NCEP-ATP III) [41] were diagnosed as positive for MetS: waist circumference>88 cm; triglycerides≥150 mg/dL; HDL cholesterol<50 mg/dL; blood pressure≥130/85 mmHg or under therapy; fasting glucose≥100 mg/dL or under therapy.

### Anthropometry

The anthropometric data included weight, height, body mass index (BMI = weight/height^2^) and waist circumference (WC). Weight and height were determined with a standard balance beam scale (max. 150 kg, 0.1 kg accuracy) and portable wall anthropometer (0.1 cm accuracy), respectively, with patients wearing lightweight clothes and no shoes. BMI was classified according to the system used by the World Health Organization (2002): lower than 25 kg/m^2^ was defined as normal, from 25–29.9 kg/m^2^ as overweight, above 30 kg/m^2^ as obesity. Waist circumference was measured at the midpoint between the lowest rib and the top of the iliac crest. The patients were advised to remain in the orthostatic position and the reading was performed at the moment of exhalation. This measurement was performed by a single evaluator (Nahas, EAP). Any WC exceeding 88 cm was considered elevated [41].

### Laboratory tests

Blood samples were collected from each subject, after 12 hours of fasting. After centrifugation to remove the clot, samples underwent biochemical analysis immediately and a serum aliquot was frozen and kept at −80°C for cytokine determinations. Triglycerides (TG), total cholesterol (TC), HDL, glucose, and C-reactive protein (CRP) measurements were processed by an automated analyzer, Model Vitros 950, by the colorimetric dry-chemistry method (Johnson & Johnson, Rochester, NY, USA). Low density lipoprotein (LDL) cholesterol was calculated using the Friedewald formula in which total cholesterol is subtracted from the sum of HDL cholesterol and triglycerides, with the result being divided by five, which shows a usage limitation when TG values exceed 400 mg/dL. The values considered to be optimal were: TC<200 mg/dL, HDL>50 mg/dL, LDL<100 mg/dL, TG<150 mg/dL, glucose<100 mg/dL and CRP<1.0 mg/dl. Insulin was quantified using Immulite System^®^ (DPC^®^, USA), which uses a solid phase chemiluminescence immune-assay and assessed in the designated automatic analyzer for the quantitative reading. The normality rate ranged from 6.0 to 27.0 µIU/ml. To evaluate insulin resistance (IR), we used a method that was based on statistical measurement of two plasma components (insulin and fasting glucose). HOMA-IR (Homeostasis Model Assessment-Insulin Resistant) was calculated using the formula: Insulin mU/ml×fasting glucose mg/dl/405. IR was defined as HOMA-IR>2.7 [42].

Tumor necrosis factor alpha (TNF-α) and interleukin 1-β (IL-1β), IL-6 and IL-10 were assayed using commercial ELISA kits (High Sensitivity Human TNF, High Sensitivity Human IL-1β, High Sensitivity Human IL-6, High Sensitivity Human IL-10, Quantikine Kits, R&D^®^ Systems, Minneapolis, MN, USA) according to manufacturer instructions. All measurements were performed at the same time to avoid inter-assay variation. Intra- and interassay coefficients of variation were <7%. The analytical High sensitivity was 0.191 pg/ml for TNF-α, 0.14 pg/ml for IL-1β, 0.11 pg/ml for IL-6, and 0.17 pg/ml for IL-10.

TLR-2 and TLR-4 expressions in peripheral blood were evaluated by RNA extraction and subsequent PCR-RT (polymerase chain reaction in real time). Total RNA was purified from heparinized blood, extracted with the Trizol system and subsequently treated with DNAse to prevent false positive results due to amplification of contaminating genomic DNA. Total RNA concentrations were determined from the absorbance values of the samples at 260 nm. The cDNA was synthesized from 1 µg total RNA using primers and kits for initiating cDNA synthesis (Table 1). The cDNA was subjected to quantitative real time PCR using ABI PRISM 7000. Probes and primers specific for human TLR-2 and TLR-4 were used according to manufacturer's instructions (Integrated DNA Technologies, Inc., USA).

**Table 1:** Sequence of primers used in real-time Polymerase Chain Reaction (PCR).

Gene	Sense (5′-3′) – Forward	Anti-Sense (3′-5′) – Reverse
TLR-2	GCCAAAGTCTTGATTGATTGG	TTGAAGTTCTCCAGCTCCTG
TLR-4	TGGATACGTTTCCTTATAAG	GAAATGGAGGCACCCCTT

TLR, *Toll-Like Receptor.*

### Statistical Analysis

The independent variables were the expression of TLR-2 and TLR-4 and the production of IL-1β, IL-6, TNF-α and IL-10. The dependent variables were the diagnostic markers of the MetS: waist circumference (WC), systolic and diastolic blood pressure (SBP and DBP), glucose, insulin resistance (IR), triglycerides, HDL and the presence of MetS. The relationship model under test assumes that the expression of TLR-2 and TLR-4 and cytokine production TNF-α, IL-1β, IL-6 e IL-10 present relationship with markers of MetS. The association between the expression of TLR-2 and TLR-4 and the presence or absence of MetS was analyzed by the chi-square test. The linear regression model was used to assess the association between the expression of TRL-2 and TRL-4 and the level of cytokines with the presence of metabolic syndrome adjusted by age and time since menopause (numeric value), BMI (categorical value), smoking status (as categorical value), and status of physical activity (categorical value). Cytokines values were divided into quartiles, highest percentile (P75) of pro-inflammatory cytokines (IL-1β IL-6, TNF-α) concentrations and lowest percentile (P25) of IL-10 concentration. The statistical tests were bilateral, adopting a 5% level of significance. All analyses were performed using the Statistical Analysis System (SAS) 9.2 program.

## Results

Clinical characteristics of the study subjects are shown in Table 2. The median age of subjects was 54 years and time since menopause 5 years; 37.6% of the women were classified as obese (IMC≥30 kg/m^2^) and 53.4% with increased waist circumference (>88 cm), showing central body fat distribution. The percentage of sedentary women was 83.9%, with only 16.1% reporting regular physical exercise (walks) at least five times a week. According to NCEP/ATPIII guidelines, 27.6% of the study participants were diagnosed with MetS, and 25.7% were insulin resistant by HOMA-IR. The expression of TLR-2 mRNA was demonstrated in 32.5%, and TLR-4 mRNA in 20.6%, of the participants (Table 2).

**Table 2:** Descriptive characteristics of 311 postmenopausal women.

Variables	Values
Age, y (median, interquartile)	54 (49–58)
Menopause age, y (median, interquartile)	48 (45–50)
Time since menopause, y(median, interquartile)	5 (2–10)
BMI≥30 kg/m^2^ (n,%)	117 (37.6)
Waist Circumference>88 cm (n, %)	166 (53.4)
Current Smoking (n,%)	51 (16.4)
Sedentary (n %)	261 (83.9)
Presence of MetS (n,%)	86 (27.6)
HOMA-IR>2.7 (n,%)	80 (25,7)
Total Cholesterol≥200 mg/dL (n,%)	135 (43.4)
HDL<50 mg/dL (n,%)	110 (35.4)
LDL>100 mg/dL (n,%)	227 (73.0)
Triglycerides≥150 mg/dL (n,%)	111 (35.7)
Glucose≥100 mg/dL (n,%)	56 (18.0)
IL-1ß, pg/mL (median, interquartile)	0.47 (0.21–0.91)
IL-6, pg/mL (median, interquartile)	0.43 (0.26–0.71)
TNF-a, pg/mL (median, interquartile)	1.27 (0.49–2.80)
IL-10, pg/mL (median, interquartile)	3.35 (2.23–5.18)
TLR-2 expressed (n,%)	94 (32.7)
TLR-4 expressed (n,%)	59 (20.6)

y, years; n, number; BMI, body mass index, AMI, acute myocardial infarction, HOMA-IR, Homeostasis Model Assessment-Insulin Resistant, HDL, high density lipoprotein, LDL, low density lipoprotein, CRP, C-reactive protein, IL, interleukin; TNF, tumor necrosis factor, TLR, Toll Like Receptor.

There was no association between the expression of TLR-2 and TLR-4 and the presence or absence of MetS (Table 3). In adjusted linear regression no association was identified (TLR-2 expressed, OR 1.21; 95% CI, 0.64–2.29, and TLR-4 expressed, OR 1.11; 95% CI, 0.51–2.40). In the analysis of cytokine values with the presence or absence of MetS, only the lower quartile of IL-10 was associated with the MetS (Table 4).

**Table 3:** Association between the expression of Toll-like receptors and the metabolic syndrome in postmenopausal women with (positive, n = 86) and without (negative, n = 225) metabolic syndrome.

Receptors	Metabolic Syndrome		*P* value^a^
	negative positive		
**TLR-2**			0.480
Expressed (n = 94)	70 (74%)	24 (26%)	
Not Expressed (n = 193)	136 (70%)	57 (30%)	
**TLR-4**			0.576
Expressed (n = 228)	161 (71%)	67 (29%)	
Not Expressed (n = 59)	45 (76%)	14 (24%)	

Values expressed as number and percentage in parentheses.

TLRs, Toll like receptor 2 and 4.

a Significant difference *P*<0.05 (Chi-Square).

**Table 4:** Association between cytokines values (in quartiles) and the presence or absence of metabolic syndrome in postmenopausal women.

Cytokines	OR^a^	95% CI	*P* value^b^
IL-1β, P75	0.98	0.41–2.32	0.96
IL-6, P75	1.24	0.59–2.61	0.55
IL-10, P25	2.43	1.06–5.57	0.03
TNF-α, P75	0.78	0.39–1.54	0.47

IL, interleukins (pg/ml); TNF tumor necrosis factor (pg/ml); P75, percentile 75; P25, percentile 25.

a Adjusted for age, time since menopause, body mass index, smoking and physical activity.

b Significant difference p<0.05 (linear regression model).

Evaluating the association between TLR-2 and TLR-4 expression and cytokine values, a greater production of IL-6 was associated with TLR-2 and TLR-4 expression, and greater production of TNF-α and lower production of IL-10 were associated only with TLR-2 expression (Table 5). There was no association between cytokines values and markers of MetS (Table 6).

**Table 5:** Association between the expression of Toll-like receptors (TLR-2/4) and the production of cytokines (in quartiles) in 311 postmenopausal women.

	OR^a^	95%IC	P value^b^
**TLR-2**			
IL-1β, P75	1.99	0.98–4.04	0.06
IL-6, P75	2.21	1.14–4.27	0.01
IL-10, P25	2.03	1.01–4.10	0.04
TNF-α, P75	1.88	1.01–3.54	0.04
**TLR-4**			
IL-1β, P75	2.13	0.85–5.37	0.10
IL-6, P75	2.95	1.26–6.90	0.01
IL-10, P25	1.15	0.52–2.54	0.71
TNF-α, P75	0.83	0.42–1.65	0.60

TLRs, Toll like receptor 2 and 4; IL, interleukins (pg/mL); TNF tumor necrosis factor (pg/mL); P75, percentile 75; P25, percentile 25.

a Adjusted results for age, time since menopause, body mass index and physical activity.

b Significant difference p<0.05 (linear regression model).

**Table 6:** Association between cytokines values (in quartiles) and markers of the metabolic syndrome in 311 postmenopausal women.

	SBP	DBP	Glucose	HDL	TG	WC
IL-1β, P75	1.40 (0.68–2.91)	0.81 (0.35–1.90)	0.98 (0.45–2.15)	1.73 (0.89–3.35)	0.82 (0.43–1.55)	1.27 (0.50–3.21)
IL-6, P75	0.60 (0.30–1.18)	0.49 (0.22–1.08)	0.67 (0.32–1.39)	1.26 (0.70–2.25)	1.14 (0.66–1.96)	0.90 (0.39–2.04)
IL-10, P25	0.91 (0.48–1.72)	1.77 (0.91–3.42)	1.72 (0.91–3.26)	0.66 (0.37–1.17)	1.09 (0.64–1.87)	0.94 (0.44–2.05)
TNF-α, P75	0.78 (0.40–1.52)	0.53 (0.24–1.16)	0.89 (0.44–1.82)	0.83 (0.46–1.49)	0.94 (0.54–1.62)	1.32 (0.57–3.06)

IL, interleukins (pg/mL); TNF tumor necrosis factor (pg/mL); SBP, systolic blood pressure; DBP, diastolic blood pressure; HDL, high density lipoprotein; WC, waist circumference; TG, triglycerides; P75, percentile 75; P25, percentile 25. Data are presented as Odds ratio and confidence interval (95%), and adjusted for age, time since menopause, body mass index and physical activity.

## Discussion

In the present study, the expression of TLR-2 and TLR-4 in peripheral blood monocytes was associated with increased pro-inflammatory cytokines (IL-6 and TNF-α), without an association to biomarkers of MetS. The expression of TLR appears to be the precursor for initial activation of inflammatory mediators such as cytokines in postmenopausal women, triggering an inflammatory response. The onset of inflammation occurs by activation of cell receptors [43]. These receptors are well described as to its association with CHD risk, especially Toll-like receptors [10], [26], [29].

Studies in animal models showed that both receptors, TLR-2 and TLR-4, are related to atherosclerosis, insulin resistance and diabetes [2], [28], [44], [45], features present in the MetS. Increased expression of TLR-2 and TLR-4 was observed in monocytes from patients with angina and acute coronary syndrome [46], [47]. In the present study we observed that the majority of women did not express mRNA for TLR-2 and TLR-4 in their peripheral blood cells. However, there was an association between TLR-2 expression and IL-6 and TNFα concentrations, suggesting that women who expressed more TLR-2 produced more cytokines. Moreover, there was an association between TLR-4 expression and IL-6 concentration.

In agreement with our results, previous studies indicated that activation of TLR-2 plays a central role in the regulation of vascular inflammation, by leading to the induction of proinflammatory cytokines [16]. The activated TLRs, particularly TLR-2 and TLR-4, regulate the induction of pro-inflammatory cytokines and oxidative burst [2], [30]. However, there are no specific previous data on this in postmenopausal women with or without MetS, with which to compare our results. MetS is defined by a set of risk factors that include abdominal obesity, dyslipidemia, hypertension and dysglycemia [1]. The MetS has a high incidence in postmenopausal and confers increased risk for CVD and diabetes [38], [48]. While MetS is considered a proinflammatory state, there is a paucity of data on cellular inflammation and about the signaling pathway of inflammation in postmenopausal women. TLRs are classical pattern recognition receptors of the innate immune response. Jialal et al. examined the expression of TLR-2 and TLR-4 in 81 male and female patients with or without MetS, aged 21–71 years. The authors found that both TLR2 and TLR4 expression and activity were increased in the monocytes of patients with MetS and could contribute to an increased risk for diabetes and CVD [38].

Ghanim et al. investigated whether peripheral blood mononuclear cells from 16 obese subjects were in a proinflammatory state when compared with 16 normal-weight controls. The authors showed that mononuclear cells in obese individuals are in a proinflammatory state with an increase in intranuclear factor *kappa* B (NF-kB) activity and that insulin resistance is a function of inflammatory mediators [49]. These data reinforce that activation of TLRs induces signaling pathway for NF-kB for the transcription of genes involved in cytokine production [50]–[52]. In the present research we did not demonstrate differences in the expression of TLR-2 and TLR-4 between women with and without MetS. This may result from the low frequency of MetS (27.6%) in the study population consisting of healthy women with a median age of 54 years and five years postmenopausal, and only 37.6% who were obese. Although there is large amount of data on inflammation and obesity in the MetS phenotype, not all obese patients have an elevated metabolic risk; 31.7% of obese people have a low risk metabolic phenotype [53].

In the present study, MetS was associated with a low percentile for IL-10. The effects exerted by IL-10 probably accentuate the anti-inflammatory character of the vascular system by inhibiting the production of pro-inflammatory cytokines [54]. It has been reported that circulating levels of IL-10 are elevated in obese and low levels of IL-10 are associated with MetS [55]. These findings demonstrate that IL-10 is elevated in obesity and this may promote an inhibition of the production of pro-inflammatory cytokines [55]. Interestingly, studies show an intriguing association between the low concentration of serum IL-10 to events related to CVD and MetS [56], [57]. There are lower serum IL-10 concentrations in patients with unstable angina compared with those who had chronic angina. It was hypothesized that decreased IL-10 levels can contribute to atheromatous plaque instability [56]. There are no studies in postmenopausal women to compare our results.

Wong et al. investigated variations in a panel of cytokines over 18 months and their relation to CVD risk factors and weight loss. Data were obtained from the Woman On the Move through Activity and Nutrition (WOMAN) Study, a randomized clinical trial investigating the effect of nonpharmacologic interventions on subclinical atherosclerosis among 290 overweight postmenopausal women, aged 52–62 years. Most of the cytokines were detectable in a majority of the samples but with large individual variations. The traditional risk factors for CHD, such as age, dyslipidemia, waist circumference and RI values were associated with IL-1, IL-6 and TNF-α. Weight loss was associated with decreased levels of IL-1, IL-6 and CRP. The large and unexplained variability in cytokine levels is probably at least partially due to genetic-environmental interactions [58]. On the other hand, changes in lifestyles (physical activity, decreased body weight) appear to be the precursor to decreases in IL-10 in obese women without MetS [57], [59].

There was no association between cytokine values and biomarkers of MetS in our study. These data are in agreement with previous studies [60]. In a nested case-control study 800 women with incident hypertension and 800 matched controls were evaluated, each group with equal numbers of white and black women within the Women's Health Initiative Observational Study. Higher CRP and IL-6 concentrations were associated with increased risk of hypertension in both white and black women. However, after adjustment for measures of adiposity, there was no significant association of CRP, IL-6, IL-1β, or TNF-α with incident hypertension in women [61]. In another study, researchers showed no difference in cytokine values between postmenopausal women with and without MetS. Nevertheless, those with abdominal obesity and hypertension exhibited a significant increase in levels of IL-6, regardless of MetS [62]. However, in a study comparing 82 women divided into two groups according to their hormonal status: pre- (n = 34) and postmenopausal women (n = 48) no significant differences were observed between IL-2, IL-4, IL-5, IL-10, IL-12 and IFN-γ, but, IL-6, TNF-α and IL-18 levels were significantly higher in postmenopausal women, especially those positive for components for the MetS [60].

There are certain limitations to a cross-sectional study. It restricts our ability to assess temporal relationships between the expression of TLRs and MetS and to identify causal biological mechanisms underlying this association. Secondly, the sample consisted of women seeking medical care, 78.9% of the women studied were less than 60 years of age, characteristic of the population of women with climacteric symptoms who seek our care, and low cardiovascular risk, impairing extrapolation of prevalence to the general population. On the other hand, there are no previous data on TLRs and MetS in postmenopausal women. Therefore, this study might be useful as a reasonable starting point to approach this issue. Further studies with the specific group of postmenopausal women to assess the association of TLRs and cytokines influencing the risk markers for MetS, are important, since the literature is still controversial.

## Conclusion

In the present study, the TLR-2 expression was associated with increased levels of the pro-inflammatory cytokines, IL-6 and TNF-α, and TLR-4 expression was associated with increased IL-6, without any association to biomarkers of MetS. The expression of TLR seems to be the initial precursor for activation of inflammatory mediators such as cytokines in postmenopausal women. However, the low concentrations of IL-10 may suggest an anti-inflammatory modulation in postmenopausal women with MetS.
